# Regional variation of premature mortality in Ontario, Canada: a spatial analysis

**DOI:** 10.1186/s12963-019-0193-9

**Published:** 2019-07-31

**Authors:** Emmalin Buajitti, Tristan Watson, Todd Norwood, Kathy Kornas, Catherine Bornbaum, David Henry, Laura C. Rosella

**Affiliations:** 1 Dalla Lana School of Public Health, University of Toronto, 155 College Street, Toronto, Ontario M5T 3 M7 Canada; 2 Institute for Clinical Evaluative Sciences, Room G-106, 2075 Bayview Avenue, Toronto, Ontario M4N 3 M5 Canada; 3 Public Health Ontario, 480 University Ave, Toronto, Ontario M5G 1 V2 Canada

**Keywords:** Epidemiology, Health policy, Public health, Geography, Premature mortality

## Abstract

**Background:**

Premature mortality is a meaningful indicator of both population health and health system performance, which varies by geography in Ontario. We used the Local Health Integration Network (LHIN) sub-regions to conduct a spatial analysis of premature mortality, adjusting for key population-level demographic and behavioural characteristics.

**Methods:**

We used linked vital statistics data to identify 163,920 adult premature deaths (deaths between ages 18 and 74) registered in Ontario between 2011 and 2015. We compared premature mortality rates, population demographics, and prevalence of health-relevant behaviours across 76 LHIN sub-regions. We used Bayesian hierarchical spatial models to quantify the contribution of these population characteristics to geographic disparities in premature mortality.

**Results:**

LHIN sub-region premature mortality rates ranged from 1.7 to 6.6 deaths per 1000 per year in males and 1.2 to 4.8 deaths per 1000 per year in females. Regions with higher premature mortality had fewer immigrants and higher prevalence of material deprivation, excess body weight, inadequate fruit and vegetable consumption, sedentary behaviour, and ever-smoked status. Adjusting for all variables eliminated close to 90% of geographic variation in premature mortality, but did not fully explain the spatial pattern of premature mortality in Ontario.

**Conclusions:**

We conducted the first spatial analysis of mortality in Ontario, revealing large geographic variations. We demonstrate that well-known risk factors explain most of the observed variation in premature mortality. The result emphasizes the importance of population health efforts to reduce the burden of well-known risk factors to reduce variation in premature mortality.

**Electronic supplementary material:**

The online version of this article (10.1186/s12963-019-0193-9) contains supplementary material, which is available to authorized users.

## Introduction

Dying prematurely—before an expected or average age of death—is a signal of unfulfilled life expectancy. Many premature deaths are considered to be avoidable through appropriate prevention or early and effective treatment [1, 2]. Analyses of premature mortality, a robust indicator of population health and health system performance, can be meaningful to health system planning and evaluation [3–5]. Geographic variation in premature mortality can indicate missed opportunities within the health system. Monitoring areas with high premature mortality, and elucidating the determinants of geographic inequalities, can support efforts to ensure effective and equitable delivery of public health and health care services.

Analysis of premature mortality in Ontario’s 14 large health service regions, known as Local Health Integration Networks (LHINs), revealed large geographic disparities in premature mortality [6]. Furthermore, gaps between regions were shown to have increased between 1992 and 2015 [6]. These large variations signal where the health system may be falling short or where upstream determinants can be better addressed. Ontario’s Patients First: Action Plan for Health Care acknowledged that access to health care varies across the province, and committed the provincial government to improving health system access and delivery [7]. To support this goal, 76 LHIN sub-regions were formalized in 2017 to serve as the focal point for local population-based planning, performance improvement and service integration [8]. These sub-regions were developed in consultation with community, physician, and government stakeholders, and emphasize the importance of targeting population health efforts at a smaller scale to better meet the needs of diverse populations [8].

Analysis of population health outcomes across the new LHIN sub-regions has not yet been done. Furthermore, no spatial analysis of Ontario mortality has been carried out at any small-area level. This gap reflects the complexity of working with small-area rates, which are often unstable and spatially autocorrelated, in part due to small population counts [9]. Rigorous small-area analysis thus requires application of dedicated spatial analytic techniques. The added benefit of these techniques is that they allow multilevel analysis of individual-, population-, and system-level effects to fully characterize the drivers of population health inequalities [10, 11].

We used population-based mortality data to identify small-area disparities in Ontario premature mortality, using the newly formed LHIN sub-regions as the geographic unit of analysis. Further, we integrated linked demographic and behavioural data to quantify the impact of underlying population-level characteristics on premature mortality and to investigate how fully these population-level differences can explain patterns of premature mortality.

## Methods

### Data sources

We identified premature deaths from the Ontario Registrar General’s death file (ORG-D), a comprehensive database of population mortality data linked at ICES [12]. ORG-D captures all deaths registered in Ontario and is linked via the Registered Persons’ Database (RPDB), which includes records for all Ontario residents that have received a health card for the province’s single-payer health coverage [12]. The linkage between ORG-D and RPDB has been described in detail elsewhere [13]. Between 2011 and 2015, the linkage rate for ORG-D records was greater than 99%. We assigned each death to one of the 76 sub-regions using postal code information from RPDB.

Linked data from the census-derived Ontario Marginalization Index (ON-Marg) were used to measure material deprivation, a proxy of socioeconomic status [14]. ON-Marg assigned individuals to provincial quintiles of material deprivation, based on area-level indicators of socioeconomic status measured by the Canadian Census and Statistics Canada income tax returns [15].

Immigration, Refugees and Citizenship Canada’s (IRCC) permanent resident database was used to identify immigrants in each sub-region [13]. IRCC captures landed immigrants who arrived in Ontario between 1985 and 2012.

We obtained behavioural risk factor data from the *Cancer Risk Factors Atlas of Ontario* [16]. That publication used seven pooled cycles (2000–2014) of the Canadian Community Health Survey (CCHS) to produce small-area prevalence estimates for modifiable risk factors relevant to chronic disease prevention. Detailed survey methodology for the CCHS is available elsewhere [17].

### Primary outcome

We defined our primary outcome, adult premature death, to include all deaths registered in ORG-D between 2011 and 2015 for decedents between the ages of 18 and 74. In doing so, we intended to capture adult deaths from all causes that occurred before full life expectancy was achieved. Specifically, this age range is based on the cut-off used by the Canadian Institute for Health Information [18] and is consistent with definitions of premature mortality in other industrialized nations [19–21]. Between 2011 and 2015, we identified 163,920 linked records of adult premature death.

### Other variables

Population-level characteristics were measured to assess the influence of key sociodemographic and behavioural risk factors on premature mortality outcomes across Ontario. These characteristics were grouped into two categories: demographic variables, which included material deprivation and immigrant population size, and health-relevant behavioural factors, which included current alcohol consumption, excess body weight, inadequate fruit and vegetable consumption, sedentary behaviour, and ever-smoked status. All covariates were measured as sex-specific prevalence estimates at the sub-region level. For all statistical models, prevalence estimates were normalized to *z*-scores based on the distribution across all 76 LHIN sub-regions.

Material deprivation was operationalized as the proportion of each LHIN sub-region’s population in the highest provincial quintile of material deprivation. Material deprivation, which describes the likelihood that an individual cannot afford or attain necessary goods and services, is a measure of low socioeconomic status [22]. Immigrant population size was defined as the percent of residents for each LHIN sub-region that were registered as immigrants with the Canadian government.

All behavioural risk factors were measured in the 12-and-older population, according to pre-existing CCHS variable definitions [16, 17]. Current alcohol consumption was defined as the percent of LHIN sub-region residents who reported drinking alcohol in the 12 months prior to survey date. Excess body weight was defined as the percent of residents categorized as overweight (25 ≤ BMI < 30) or obese (BMI ≥ 30) based on calculations from self-reported height and weight measurements. Inadequate fruit and vegetable consumption was measured as the percent of residents who reported daily consumption of fruits and vegetables fewer than five times. Sedentary behaviour was defined as the percent of sub-region residents who spent more than 15 hours per week watching television or videos, or on the computer. Finally, ever-smoked status was defined as the proportion of sub-region residents who reported being a current or former smoker (either daily or occasional).

### Statistical analysis

We calculated 5-year (2011–2015) annualized adult premature mortality rates for all 76 sub-regions. We report sex-specific rates, which have not been age-standardized, as deaths per 1000 per year. We also calculated “excess” deaths in each sub-region as the total number of premature deaths that would have been avoided if that sub-region experienced the premature mortality rate of North West Mississauga (which had the lowest 2011–2015 rate for males and females). Excess death counts reflect the absolute burden of premature mortality and have been included to contextualize the mortality rates observed in each LHIN sub-region.

We used a Bayesian hierarchical approach to model the effect of sub-region on premature mortality, adjusting sequentially for demographic and behavioural covariates. Bayesian hierarchical models are commonly used in small-area disease mapping, because they stabilize small-area rate estimates while accounting for patterns of spatial autocorrelation that violate the assumptions of other approaches [10, 11]. Specifically, the Besag-York-Mollié model [23] our analysis is based on has been found to be a highly robust approach to small-area disease mapping [24].

We fit two-level Bayesian hierarchical Poisson models. In the first level, the observed number of premature deaths in a given sub-region is decomposed to the age-standardized expected number of deaths in that region multiplied by a sub-region-specific standardized mortality ratio (SMR). We calculated the expected deaths for each sub-region using sex- and age-specific premature mortality rates for all Ontario. Age-standardizing the expected death counts accounts for differences in age structure across LHIN sub-region populations which may influence model estimates.

The second level contains the covariates which determine a region’s mortality ratio, and includes both fixed and random effects. We used fixed effects to represent the effect of population-level demographic and behavioural covariates, and random effects to represent the direct system-level sub-region effect. The random effects contain both spatially structured and unstructured components, which vary independently. The full model specification and implementation is described in Additional file 1.

We specified three age-standardized, sex-stratified models. The first, unadjusted model, contained only random effects. The second, demographics-only model, adjusted for population material deprivation and immigrant proportion as of 2012. The third, demographics plus behaviours model, introduced five population-level behaviours: alcohol consumption, excess body weight, inadequate fruit and vegetable consumption, sedentary behaviour, and ever-smoked status. We fit models using Markov chain Monte Carlo (MCMC) sampling methods with non-informative priors.

From each model, we extracted relative risk estimates and 95% credible intervals for all fixed-effect covariates, and adjusted SMR estimates for each individual sub-region. To compare models, we calculated the global variance in SMR estimates. This global variance statistic quantifies sub-region variation in premature mortality, after adjusting for model covariates. It can be used to calculate how much geographic disparity is explained by population differences.

We also calculated the spatial component of variance, which ranges from 0 to 1 and is defined as the proportion of random effect variance attributable to the spatial random effect [25]. A spatial component of variance approaching 1 suggests unexplained spatial variance.

To ensure the appropriateness of our spatial models, we repeated our analysis using generalized linear mixed models with no structured spatial component. These model results were compared to output from the Bayesian models. The residual terms from these models were tested for spatial autocorrelation using a Moran’s *I* statistic.

### Software

Descriptive statistics were prepared using SAS software version 9.4 [26]. Models were fit in R version 3.3.4 [27], using the package R2WinBUGS [28] to call Bayesian statistical program WinBUGS version 1.4.3 [29].

## Results

### Premature mortality rates

**Fig. 1:**
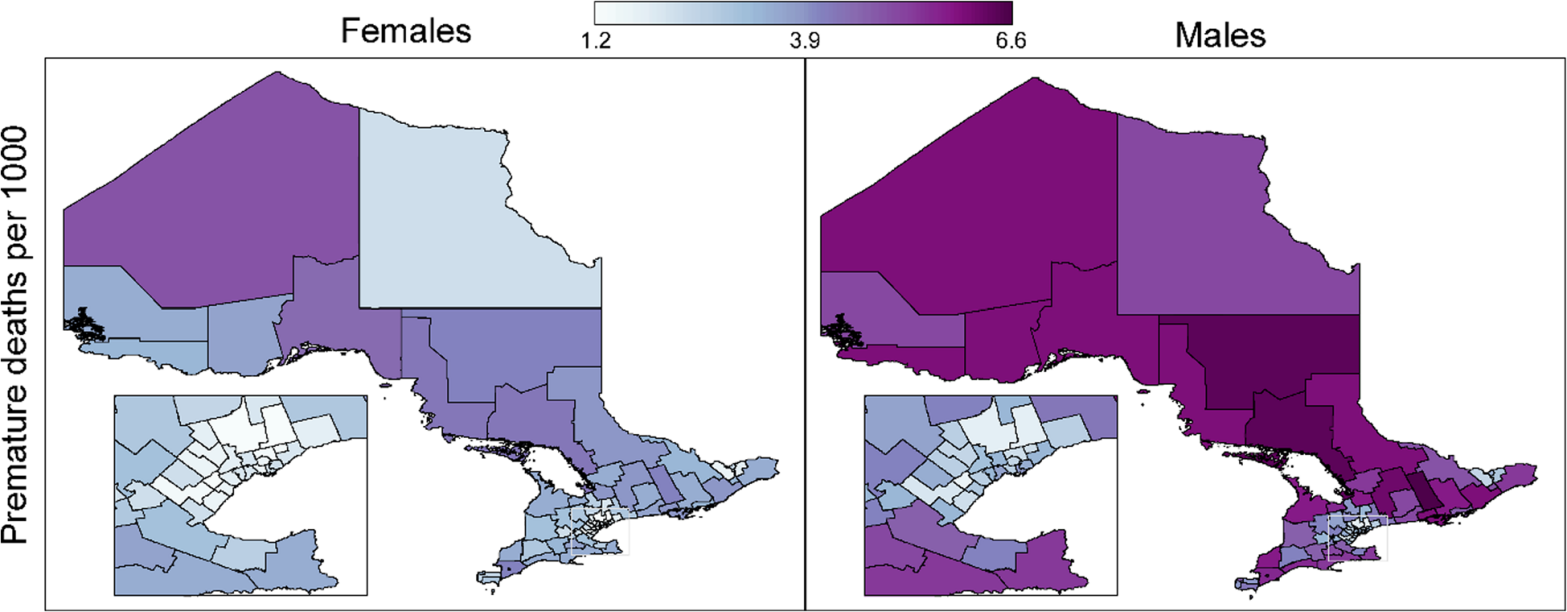
Premature mortality rates (deaths per 1000 per year) by sex and LHIN sub-region, Ontario, 2011–2015

Adult premature mortality rates by LHIN sub-region are mapped in Fig. 1. Among males, the province-wide mortality rate was 3.78 deaths per 1000 per year, compared to 2.50 deaths per 1000 per year among females. A total of 163,920 premature deaths were recorded between 2011 and 2015. Excess deaths by LHIN sub-region are mapped in Additional file 1: Figure S1.

Large geographic disparities were seen in both males and females. Mortality rates ranged from 1.71 (North West Mississauga) to 6.57 (Rural Hastings) premature deaths per 1000 per year in males, and from 1.22 (North West Mississauga) to 4.83 (Northern) premature deaths per 1000 per year in females. In each sub-region, premature mortality rates were higher in males than in females.

In males and females, low premature mortality rates were concentrated in south-central Ontario with a secondary cluster in southeast Ontario near the eastern (Québec) border. These clusters represent the metropolitan areas surrounding Toronto and Ottawa, both of which are large, urban centres. Premature mortality rates were comparatively higher throughout the rest of the province, particularly in the north.

### Population-level risk factors

Table 1 shows the distribution of measured population risk factors. Regions have been grouped into three categories by premature mortality rate. The categories were assigned according to the 95% credible interval estimated for each region’s SMR by the unadjusted model. We classified regions with 95% credible intervals entirely above 1 (the mortality ratio for Ontario) as “Higher than Ontario”, those with intervals entirely below 1 as “Lower than Ontario”, and those with intervals spanning 1 as “Neither Higher nor Lower.”

**Table 1:** Baseline characteristics by mortality risk group, Ontario, 2011-2015

Variable	Sex	Mortality risk group; prevalence of risk factor (%)			
		Lower than Ontario	Neither higher nor lower	Higher than Ontario	Overall
No. of LHIN sub-regions	–	25	12	39	76
2013 population	M	2,478,453	677,688	2,024,960	5,181,101
	F	2,581,117	673,631	2,014,520	5,269,268
Premature mortality rate (deaths per 1000 per year)	M	2.68	3.72	5.16	3.78
	F	1.77	2.46	3.45	2.50
Demographics					
Percentage in highest quintile material deprivation	M	24.0	20.5	21.7	22.6 (SD = 16.6)
	F	24.5	20.3	22.3	23.1 (SD = 16.5)
Percentage of immigrants	M	32.1	14.3	6.0	19.6 (SD = 14.7)
	F	32.8	15.0	6.2	20.4 (SD = 15.0)
Behaviours^a^					
Percentage of current alcohol consumption	M	77.7	80.8	79.5	78.8 (SD = 2.4)
	F	67.9	76.1	74.0	71.3 (SD = 5.4)
Percentage of excess body weight (overweight/obese)	M	51.2	53.6	59.4	54.7 (SD = 5.1)
	F	37.4	38.8	46.3	41 (SD = 5.5)
Percentage of inadequate vegetable and fruit consumption	M	74.9	75.2	78.9	76.5 (SD = 3.2)
	F	62.1	61.2	65.1	63.1 (SD = 3.2)
Percentage of sedentary behaviour	M	53.9	54.4	55.2	54.5 (SD = 2.4)
	F	46.2	46.5	49.9	47.6 (SD = 3.0)
Percentage of ever smokers	M	12.0	28.2	49.1	28.6 (SD = 22.5)
	F	15.9	37.8	48.0	30.9 (SD = 18.9)

^a^CCHS-derived variable from *Cancer Risk Factors Atlas of Ontario* (2017)

Thirty-nine sub-regions were classified as “Higher than Ontario” according to this approach, with 25 “Lower than Ontario” and 12 “Neither Higher nor Lower”. In general, sub-regions with higher premature mortality had fewer immigrants and higher prevalence of excess body weight, inadequate fruit and vegetable consumption, sedentary behaviour, and ever-smoked status.

### Model output

The results from the Bayesian hierarchical Poisson models are presented in Table 2. In the final (demographics plus behaviours) model, four of seven modelled risk factors had a statistically significant effect on premature mortality (*p* ≤ 0.05) for males and females: material deprivation, immigrant population, alcohol consumption, and ever-smoked status. Increased prevalence of high material deprivation and ever-smoked status was associated with increased premature mortality risk, while large immigrant populations and higher prevalent alcohol consumption were associated with decreased mortality risk. The direction and approximate size of all effects were consistent between sexes.

**Table 2:** Bayesian hierarchical Poisson models for premature mortality, Ontario, 2011-2015

Variable	RR (95% credible Interval)^a^					
	Unadjusted model		Demographics only		Demographics and behaviours	
	Males	Females	Males	Females	Males	Females
Percentage of highest quintile material deprivation	–	–	1.17 (1.11–1.24)	1.13 (1.07–1.19)	1.1 (1.04–1.16)	1.08 (1.02–1.15)
Percentage of immigrants	–	–	0.74 (0.69–0.79)	0.79 (0.74–0.85)	0.78 (0.68–0.87)	0.79 (0.69–0.93)
Percentage of current alcohol consumption^b^	–	–	–	–	0.92 (0.85–0.98)	0.89 (0.8–0.99)
Percentage of excess body weight (overweight/obese)^b^	–	–	–	–	0.99 (0.9–1.07)	1.05 (0.95–1.18)
Percentage of inadequate vegetable and fruit consumption^b^	–	–	–	–	0.94 (0.87–1.02)	0.95 (0.89–1.02)
Percentage of sedentary behaviour^b^	–	–	–	–	1.04 (0.99–1.09)	1.02 (0.96–1.09)
Percentage of ever smokers^b^	–	–	–	–	1.21 (1.11–1.32)	1.16 (1.03–1.3)
Explained regional variation, %	–	–	*80.0*	*79.2*	*89.8*	*88.7*
Spatial component of variance	*0.761*	*0.751*	*0.622*	*0.604*	*0.608*	*0.570*

^a^All risk ratio estimates are for a 1 standard deviation increase in the parameter of interest

^b^CCHS-derived variable from *Cancer Risk Factors Atlas of Ontario* (2017)

Compared to the unadjusted model, adjusting for socioeconomic status and immigrant population size reduced sub-region variation in premature mortality by approximately 80%. Adjusting further for five health-relevant behaviours (current alcohol consumption, excess body weight, inadequate fruit and vegetable consumption, sedentary behaviour, and ever-smoked status) reduced the variation by an additional 10%. The final model covariates explained close to 90% of sub-regional variation in premature mortality in males and females.

For the final (demographics plus behaviours) model, the spatial component of variance was 0.608 for males and 0.570 for females. Since values closer to 1 indicate strong spatial structuring, this finding indicates that our models have residual spatial variance and suggests unresolved system-level disparities.

The results of our analysis using generalized linear mixed models are available in Additional file 1: Table S1. Estimates and confidence intervals were not meaningfully different from the Bayesian models’ output. However, the generalized linear mixed model approach assumes that spatial variation in premature mortality is randomly distributed, which as we have seen is untrue. Moran’s *I* tests for these models showed significant residual spatial autocorrelation for the random effect-only model (Moran’s *I* = 0.14, *p* = 0.001), but not for the demographics only (Moran’s *I* = 0.02, *p* = 0.25) or demographics and behaviours model (Moran’s *I* = − 0.03, *p* = 0.72).

## Discussion

### Summary of main results

We conducted the first spatial analysis of premature mortality using Ontario’s newly formalized LHIN sub-regions. We used Bayesian spatial analytic techniques to model the effect of LHIN sub-region on adult premature mortality in Ontario, adjusting for population-level demographic and behavioural characteristics. Our study showed large disparities in premature mortality at the LHIN sub-region level. Adjusting for socioeconomic status and immigrant population explained most of the geographic variation in premature mortality. Adjusting for five key behaviours further reduced sub-region variation; however, some variation remains unchanged. Additionally, we found empirical evidence that the spatial pattern of premature mortality outcomes in Ontario is not fully accounted for by population demographics or health behaviours.

### Explanation of findings

Our study revealed a clear geographic pattern of premature mortality, with low rates clustering around large urban centres in Ottawa and Toronto. This is consistent with evidence showing elevated mortality risk in rural environments [30–32]. However, it is worth noting that low-mortality clusters were not seen around any of Ontario’s smaller urban centres, such as Kingston or Sudbury. Our findings also show that population-level differences in demographic makeup have the largest sub-regional impact on premature mortality outcomes. The highest premature mortality risk was seen in sub-regions with fewer immigrants and higher material deprivation. This confirms a substantial immigrant health advantage, previously noted in Ontario for mortality [33] and cardiovascular disease [34]. It also confirms the importance of region-level socioeconomic status to population health, which has been seen consistently elsewhere [35, 36].

Together, demographic and behavioural population characteristics explained a large proportion of LHIN sub-region variations in premature mortality. However, residual spatial variation suggests that there is a systematic component underlying sub-region differences in premature mortality not explained by the population-level traits we have considered. This may be the result of system-level factors, such as access to care and risk factor control, which have been shown to modify mortality risk [37]. The implication is that population characteristics are not solely responsible for the large geographic disparities demonstrated in Ontario premature mortality. Geographic disparities may be exacerbated by system-level inequities that impact residents’ access to services and care.

### Limitations

We used population-level prevalence estimates for all measured covariates, rather than individual-level risk factor data. As such, the patterns of premature mortality identified by our analysis may be sensitive to our use of LHIN sub-regions, and the same patterns may not be seen if the analysis was repeated with a different unit of aggregation. This is an instance of the modifiable areal unit problem [38] and may limit the generalizability of our findings. Also, the CCHS prevalence estimates are designed to be representative of the non-institutionalized Canadian population aged 12 and older, which differs from our study’s target population of all Ontario adults [39]. We also were unable to examine premature deaths in childhood, due to data limitations on exposure information relevant for paediatric populations. This is an important area of future study. Finally, it is difficult to quantify the variability underlying our behavioural risk factor estimates, which may have impacted the precision of our parameter estimates in a way that is difficult to quantify. However, the approach used to generate prevalence estimates was independently validated [40].

## Conclusion

Comprehensive population-level vital statistics data reveal large geographic disparities in premature mortality in Ontario’s LHIN sub-regions. A majority of sub-regional variation can be explained by differences in population characteristics and behaviours, most notably material deprivation and immigrant status. However, residual variation shows a strong spatial component, which suggests that unexplained differences in premature mortality are systematic in nature. Future work should focus on quantifying the impact of system-level impacts (for example, access to care or behavioural interventions) on premature mortality outcomes. Additionally, greater effort is needed to understand which interventions may mitigate the negative mortality effects of low socioeconomic status and key behavioural risk factors.

## Additional file

Additional file 1: Model specification and implementation. Provides a more detailed overview of the model specification and implementation processes employed in our analyses than is described in the main manuscript. **Figure S1**. Excess premature deaths by sex and LHIN sub-region, Ontario, 2011–2015. Map showing the absolute number of excess deaths registered in Ontario LHIN sub-regions between 2011 and 2015 inclusive. **Table S1**. Results of generalized linear mixed models. Table showing the results of sensitivity analysis using generalized linear mixed models in place of Bayesian hierarchical spatial models. (PDF 1631 kb)

## Data Availability

The dataset used in this study is held securely in coded format at ICES. Although data sharing agreements prohibit ICES from making the dataset publicly available, access may be granted to those who meet the conditions for confidential access, available at www.ices.on.ca/DAS.

## Abbreviations

CCHS: Canadian Community Health Survey
IRCC: Immigration, Refugees and Citizenship Canada
LHIN: Local Health Integration Network
ON-Marg: Ontario Marginalization Index
ORG-D: Ontario Registrar General’s death file
RPDB: Registered Persons’ Database
