# Supplementary material for: Regional variation of premature mortality in Ontario, Canada: a spatial analysis
Source: Popul Health Metr. 2019 Jul 31;17:9. doi: 10.1186/s12963-019-0193-9 (PMC6670187; doi:10.1186/s12963-019-0193-9)
Supplement: Supplementary file 1 — Model specification and implementation. Provides a more detailed overview of the model specification and implementation processes employed in our analyses than is described in the main manuscript. Figure S1. Excess premature deaths by sex and LHIN sub-region, Ontario, 2011–2015. Map showing the absolute number of excess deaths registered in Ontario LHIN sub-regions between 2011 and 2015 inclusive. Table S1. Results of generalized linear mixed models. Table showing the results of sensitivity analysis using generalized linear mixed models in place of Bayesian hierarchical spatial models. (PDF 1631 kb) [file 12963_2019_193_MOESM1_ESM.pdf]

ADDITIONAL FILE 1: Model Specification and Implementation

Model Specification

We specified three hierarchical Poisson regression models of the type:

$$O_i|\theta_i, E_i \sim Poisson(\theta_i E_i)$$
$$log(\theta_i) = \mathbf{x_i^T \beta} + u_i + v_i$$

In this model, O_i represents the number of premature deaths registered in LHIN sub-region *i* between 2011 and 2015, and E_i is the number of premature deaths that would have taken place if that LHIN sub-region had experienced the overall Ontario premature mortality rate in the same period in all age groups (i.e. the age-standardized "expected" number of premature deaths in LHIN sub-region *i*). The model estimates θ_i, which is the age-standardized mortality ratio for LHIN sub-region *i*.

θi is determined by a combination of global and LHIN sub-regional effects. $\mathbf{x_i^T}$ refers to a transposed vector of region-level covariates for LHIN sub-region *i* with global parameter coefficient vector $\boldsymbol{\beta}$. u_i is an unstructured LHIN sub-regional random effect, which is assigned a non-informative normal prior. v_i is a spatially structured LHIN sub-regional random effect, which is assigned a conditionally autoregressive (CAR) prior.

The CAR prior informs the spatially structured random effect of each LHIN sub-region by borrowing information from neighbouring regions (1). This is based on the assumption that mortality ratios of LHIN sub-regions which share a border are more likely to be similar than those of regions which do not share a border (2). The spatially structured random effect then acts to preferentially smooth LHIN sub-region mortality ratio estimates towards those of neighbouring regions (1-3).

In all three model specifications, the model framework and random effects remain unchanged. Also in all models, we assigned non-informative Normal(0,100000) priors for all β parameters. However, we assigned a different number of global covariates ($\mathbf{x_i^T}$) to each model.

Model #1, the unadjusted model, contained no global covariates. Model #2, the demographics-only model, contained two global covariates: proportion of immigrants and percent of population in the highest Ontario quintile of material deprivation (a proxy for low socioeconomic status). Model #3, the fully adjusted model, contained the two demographic covariates from Model #2 plus the following five behavioural risk factors: alcohol consumption, excess body weight, inadequate fruit and vegetable consumption, sedentary behaviour and ever smoking. In all cases, models used sex-specific prevalence estimates for LHIN sub-regional risk factors.

Implementation

Six models (three sex-specific specifications) were fit using Monte Carlo Markov Chain (MCMC) sampling. In each case, we ran three parallel, overdispersed chains of 350,000 simulations each. The first 250,000 samples were discarded as a burn-in period. The remaining samples were thinned by a factor of 50. The total number of samples retained for each model was 6,000 (2,000 from each of three chains).

We assessed model convergence on the basis of formal Rubin-Gelman convergence statistics (4), and by visual inspection of the mixing of the three chains using model trace plots. On these criteria, convergence was sufficient for all models.

From each model, we extracted parameter estimates and 95% credible intervals for all parameters of interest. Parameter estimates were taken as the mean of the posterior distribution for that variable.

References

1. Chen JT. Multilevel and Hierarchical Models for Disease Mapping. In: Boscoe FP, editor. Geographic Health Data: Fundamental Techniques for Analysis. Wallingford, Oxfordshire: CAB International; 2013.
2. Lee D. A comparison of conditional autoregressive models used in Bayesian disease mapping. Spatial and Spatio-temporal Epidemiology. 2011;2(2):79-89.
3. Banerjee S, Carlin BP, Gelfand AE. Hierarchical modeling and analysis for spatial data. Boco Raton, FL: Chapman & Hall; 2004.
4. Gelman A, Rubin DB. Inference from iterative simulation using multiple sequences. Statistical science. 1992:457-72.

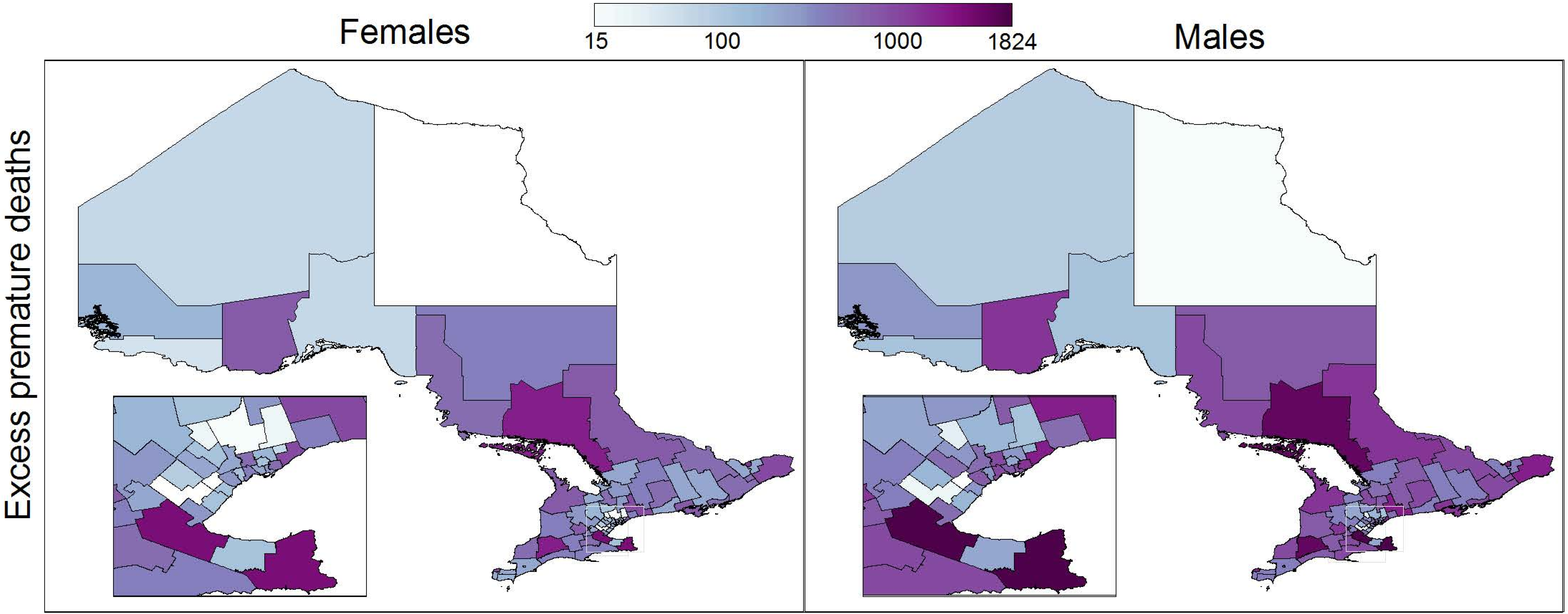
Females
15
100
1000
1824
Males
Excess premature deaths

Results of Generalized Linear Mixed Models

Generalized linear mixed models for premature mortality				
	RR (95% credible Interval)			
	Demographics only		Demographics and behaviours	
**Variable**	**Males**	**Females**	**Males**	**Females**
% highest quintile material deprivation	1.16 (1.11 - 1.21)	1.15 (1.09 - 1.20)	1.10 (1.06 - 1.15)	1.07 (1.02 - 1.12)
% immigrants	0.71 (0.68 - 0.74)	0.71 (0.68 - 0.74)	0.82 (0.76 - 0.89)	0.81 (0.72 - 0.92)
% current alcohol consumption	–	–	0.95 (0.90 - 1.00)	0.93 (0.86 - 1.00)
% excess body weight (overweight/obese)	–	–	1.03 (0.97 - 1.09)	1.09 (1.00 - 1.17)
% inadequate vegetable and fruit consumption	–	–	0.98 (0.93 - 1.04)	0.99 (0.95 - 1.04)
% sedentary behaviour	–	–	1.03 (0.99 - 1.06)	1.03 (0.99 - 1.08)
% ever smokers	–	–	1.19 (1.12 - 1.26)	1.11 (1.04 - 1.19)
Explained regional variation, %	*82.0*	*84.5*	*92.4*	*92.4*
Spatial component of variance	*N/A*	*N/A*	*N/A*	*N/A*

[1]All risk ratio estimates are for a 1 standard deviation increase in the parameter of interest.
[2]Spatial component of variance cannot be calculated for generalized linear mixed models.
